# Establishment of the menthol test as a clinical evaluation method for oxaliplatin-induced neuropathy

**DOI:** 10.20407/fmj.2021-011

**Published:** 2021-11-25

**Authors:** Yeongcheol Cheong, Hidetoshi Katsuno, Hiroshi Matsuoka, Masahiro Mizuno, Tomoyoshi Endo, Tadahiro Kamiya, Yosuke Tajima, Keigo Ashida, Yoshikazu Koide, Koji Masumori, Harunobu Sato, Tsunekazu Hanai, Kotaro Maeda, Ichiro Uyama, Junichiro Hiro, Koichi Suda

**Affiliations:** 1 Department of Gastroenterological Surgery, Fujita Health University, School of Medicine, Toyoake, Aichi, Japan; 2 Department of Gastroenterological Surgery, Fujita Health University, School of Medicine, Okazaki, Aichi, Japan; 3 Department of Surgery, Ieda Hospital, Toyota, Aichi, Japan; 4 Department of Surgery, Tottori Prefectural Central Hospital, Tottori ,Tottori, Japan; 5 Department of Advanced Robotic and Endoscopic Surgery, Fujita Health University, School of Medicine, Toyoake, Aichi, Japan; 6 International Medical Center, Fujita Health University Hospital, Toyoake, Aichi, Japan

**Keywords:** Menthol, Oxaliplatin-induced neuropathy, OPN, Oxaliplatin

## Abstract

**Objectives:**

To determine whether the tongue menthol test, which measures the cold sensation detection threshold (CDT) of the tongue, used before and after oxaliplatin administration is an objective evaluation method for oxaliplatin-induced peripheral neuropathy (OPN).

**Methods:**

The tongue menthol test was administered to patients both before and after undergoing chemotherapy containing oxaliplatin for colorectal cancer. The tongue menthol test was conducted by applying a menthol solution (a selective agonist of transient receptor potential cation channel subfamily M member 8 [TRPM8]) to the tongue and measuring the CDT.

**Results:**

The mean CDT before the first dose of oxaliplatin was 0.34% (0.005%–1%; *n*=38), and the mean CDT after the first dose was 0.32% (0.005%–1%; *n*=38). The CDT appeared to decrease after the first dose, but this difference was not significant. In patients who received five courses of oxaliplatin, changes in CDT values were compared before and after the five courses. In patients with Neurotoxicity Criteria of Debiopharm (DEB-NTC) grade 2 neuropathy, the pre-oxaliplatin administration CDT was compared between before grade exacerbation and when exacerbation occurred, and was found to decline when grade exacerbation occurred. Moreover, when the CDTs before and after administration were compared before grade exacerbation, there was a significant decrease in CDT after administration (*P*=0.04).

**Conclusions:**

By performing a menthol test in oxaliplatin-treated patients, it may be possible to objectively predict the exacerbation of peripheral neuropathy at an early stage.

## Introduction

Overall survival in advanced cancer is increasing for many cancer types, and it is thought that new anticancer drugs, such as molecular-targeted drugs and immune checkpoint inhibitors, are contributing to this increase. Oxaliplatin is a third-generation platinum drug that has an anticancer effect by inhibiting DNA synthesis. It is a key drug for colorectal cancer, gastric cancer, and pancreatic cancer.^1,2^

Although the response rate for colorectal cancer is as high as 90%, oxaliplatin-induced peripheral neuropathy (OPN) is a typical side effect that occurs at a high rate (90%)^3^ along with allergic reactions, and adversely affects the quality of life (QOL) of patients. The characteristics of OPN are cold irritant pain immediately after administration and sensory impairment in the chronic phase. As it progresses, it leads to dose reduction and the interruption of treatment.^4,5^ In addition, severely aggravated cases suffer from coasting^6,7^ (where the effects worsen even after the discontinuation of oxaliplatin) and neuropathy that can last for a lifetime even after the use of other drugs.

The major clinical problem of OPN is that medical professionals are unable to determine the degree of exacerbation of neuropathy because there are no objective evaluations of the symptoms themselves. In clinical studies of chemotherapy-induced neuropathy, the visual analog scale, numerical rating scale, and self-administered questionnaires such as patient-reported outcome, European Organisation for Research and Treatment of Cancer Quality of Life Questionnaire for Chemotherapy-Induced Peripheral Neuropathy, and Functional Assessment of Cancer Therapy/Gynecologic Oncology Group-Neurotoxicity are often used; however, there are no established objective evaluations. The Common Terminology Criteria for Adverse Events (CTCAE) and Neurotoxicity Criteria of Debiopharm (DEB-NTC) may be used, but it has been noted that their consistency with subjective evaluations is relatively low.^8^

Overexpression of the cold receptor transient receptor potential (TRP) channels has attracted attention as a possible mechanism of OPN. The most common sites of OPN are in the fingers and soles,^9^ but they also occur in the oral cavity and pharynx.^10^ TRP channels are among the ion channel-type receptors on the cell membrane, and in mammals they are composed of at least 29 types of genes and six subfamilies. Many TRP channels are activated by components of natural substances, such as plants. For example, the cold-sensitive TRP subfamily M member 8 (TRPM8) is a receptor for menthol, which is a component of mint, and is activated by this substance.^11^ Therefore, TRPM8 is likely to be useful for the evaluation of cold stimulation symptoms, especially in the acute phase of OPN.

A previous study by Kono et al.^12^ noted the presence of TRPM8 in the tongue, and first reported the menthol test (the TRPM8 stimulation test) in which a cotton swab was soaked in menthol and touched to the tongue. This test was performed before and after oxaliplatin administration in 40 healthy subjects and 36 patients with colorectal cancer.^12^ In this previous study, the lowest concentration^13^ at which a cold stimulus was felt was taken as the cold sensation detection threshold (CDT). The CDT decreased significantly after the first administration of oxaliplatin and hypersensitivity to cold stimuli was observed. In tests after several doses of oxaliplatin, the CDT decreased and hypersensitivity increased after administration. Moreover, before oxaliplatin administration, the CDT was higher than at the time of the first administration. Kono et al.^12^ suggested that these findings indicate the dulling of sensation caused by sensory impairment that is associated with increased doses of oxaliplatin. They also reported no obvious adverse events of menthol application. Together, their findings suggest that the tongue menthol test may be able to quantitatively determine OPN. However, this previous study involved measurements at two timepoints only, and there was insufficient consideration of chronic disorders, which are thought to worsen over time.

Unlike the conventional method of evaluation,^13^ the tongue menthol test may be performed before neurotoxicity becomes severe. It is therefore clinically very important to establish whether this test is suitable for determining the severity of neurotoxicity, and whether it can be used as an OPN evaluation method. The present study was designed to evaluate the usefulness of this test by assessing changes over time, which were not explored in previous studies.

Thus, the purpose of the current study was to devise a tongue menthol test, which measures the CDT by applying menthol (a selective agonist of TRPM8) to the tongue, as a method of evaluating OPN both before and after oxaliplatin administration. We also aimed to investigate whether this test was able to be used as an indicator of when to reduce the dose or discontinue administration.

## Methods

### Subjects

The inclusion criteria for the subjects were as follows:

1)  Unresectable advanced and recurrent colorectal cancer cases without chemotherapy
2)  Adjuvant chemotherapy for pathological stage II or III colorectal cancer after curative resection
  *  On the day before testing, all subjects needed to refrain from ingesting foods containing spices and stimulants
  *  Within 2 hours of testing, all subjects needed to refrain from eating, chewing gum, or using toothpaste
3)  Understood and consented to this test.

The exclusion criteria were as follows:

1)  History of chemotherapy for multiple cancers
2)  Peripheral neuropathy
3)  History of stroke or brain tumor
4)  History of severe drug hypersensitivity
5)  Pregnant or potentially pregnant women
6)  Subjects whom it was judged difficult to participate in this study because of mental illness or psychiatric symptoms
7)  Subjects whom a doctor judged not suitable for enrollment in this study.

**Figure 1:**
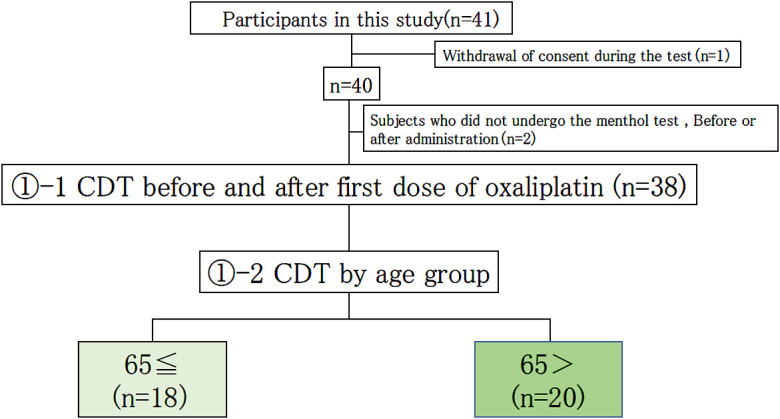
The menthol test was performed in 38 patients with colorectal cancer.

The menthol test was performed in 41 patients with colorectal cancer (Figure 1) who met the aforementioned criteria.

### Chemotherapy

Two treatment regimens, the modified FOLFOX6 (mFOLFOX6; which consists of 5-fluorouracil [5-FU], leucovorin, and oxaliplatin) and XELOX (which consists of capecitabine and oxaliplatin), were selected. For the mFOLFOX6 therapy, oxaliplatin (85 mg/m^2^) and leucovorin (200 mg/m^2^) were intravenously infused for 2 hours on day 1; this was followed by a rapid intravenous infusion of 5-FU (400 mg/m^2^) and then a portable pump of intravenous 5-FU (2400 mg/m^2^/3 days) for 46 hours. The XELOX therapy was administered on day 1 by the intravenous drip infusion of oxaliplatin (130 mg/m^2^) for 2 hours, followed by capecitabine (2000 mg/m^2^) for 14 days from day 1. Dose reduction/discontinuation was judged clinically, and the dose at the time of reduction was recorded.

### Menthol test

A 25-g package of l-menthol crystals was used. Water for the injections was added to the powdered l-menthol crystals in a closed container, and they were then dissolved in a warm water bath (50°C). Next, water for the injections was added to make a l-menthol solution in six dilution steps (0.005%, 0.01%, 0.05%, 0.1%, 0.5%, and 1%). For the test, the l-menthol solution on a cotton swab was first applied to the tip of the tongue for 5 s at each concentration. After 5 s, questions were asked. Next, the concentration was gradually increased from a low concentration. The lowest concentration at which a cold stimulus (i.e., a sensation such as cooling or burning) was felt was recorded as the CDT.

### Endpoints

There were three endpoints, as follows:

1.  Comparison of the CDT before and after the first administration of oxaliplatin.

CDT comparisons by age.

2.  Changes in the CDT in patients receiving five doses of oxaliplatin.

CDT comparisons by age.

3.  Using the DEB-NTC scale, a questionnaire about peripheral neuropathy was conducted before the oxaliplatin administration, and its association with the CDT was then analyzed.

### Statistical analysis

To evaluate the effects of oxaliplatin, the nonparametric Wilcoxon-compatible *t*-test and Wilcoxon signed rank test were used, and *P*<0.05 was considered statistically significant for all analyses. All analyses were performed using JMP Pro (ver. 15.2; SAS Institute Inc., Cary, NC, USA).

The protocol of this study was approved by the Ethics Committee of Fujita Health University (HM18-202). Before the study was conducted, written informed consent was obtained from all patients.

We conducted in accordance with the recommendations outlined in the Declaration of Helsinki.

## Results

### Endpoint 1

**Figure 2:**
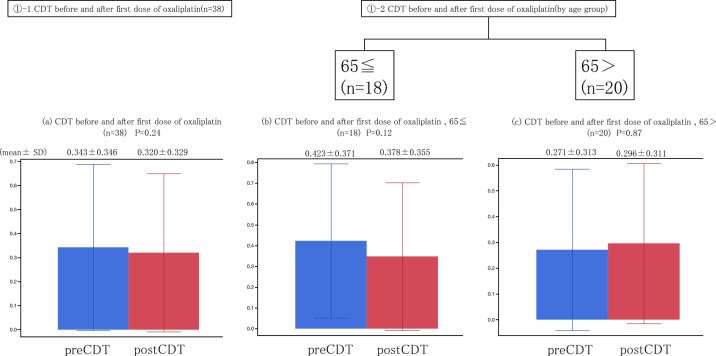
Comparison of the cold sensation detection threshold (CDT) before and after the first administration of oxaliplatin. The CDT was determined by applying menthol before and after the first oxaliplatin administration. (a) The CDT of all cases tended to decrease from 0.343±0.346 (mean±standard deviation [SD]) to 0.320±0.329 (mean±SD), *P*=0.24. (b) The CDT of individuals aged ≥65 years tended to decrease from 0.423±0.371 (mean±SD) to 0.378±0.355 (mean±SD), *P*=0.12. (c) The CDT of individuals aged <65 years tended to increase from 0.271±0.313 (mean±SD) to 0.296±0.311 (mean±SD), *P*=0.87.

Of the 41 patients who participated in this study, the mean CDT before the first oxaliplatin dose was measured in colorectal cancer patients. Two patients were excluded because their data were incomplete, and one patient was excluded because they withdrew their consent (Figure 1). The mean CDT before the first dose was 0.34% (0.005%–1%) (*n*=38), and the mean CDT after the first dose was 0.32% (*n*=38) (0.005%–1%). Although the CDT appeared to decrease from before the first dose to after the first dose, this difference was not significant (Figure 2).

### Endpoint 2

**Figure 3:**
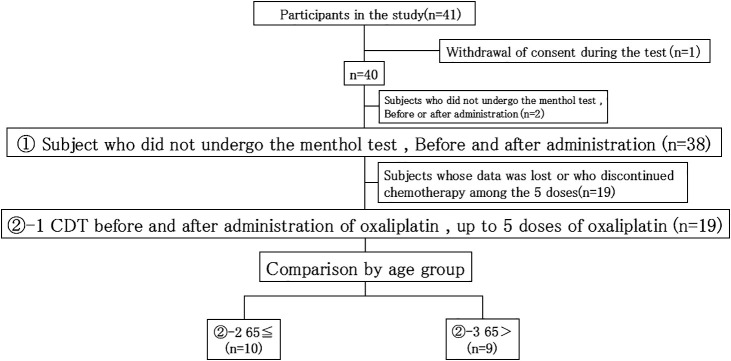
Cold sensation detection threshold (CDT) before and after the administration of oxaliplatin, with up to five doses of oxaliplatin (*n*=19)

In cases who received five doses of oxaliplatin, changes in CDT values before and after the five doses were compared. Of the 41 patients who participated in this study, 19 patients were examined (excluding those who had difficulty continuing chemotherapy, those whose data were lost, and those who withdrew their consent) (Figure 3).

As the number of oxaliplatin doses increased, the CDT also increased. Compared with the initial dose, the doses were gradually and significantly reduced in the subsequent doses (2nd dose: *P*=0.026, 3rd dose: *P*=0.002, 4th dose: *P*=0.002, and 5th dose: *P*=0.002).

The average total of the five oxaliplatin doses was 553.79 (340–650) mg/m^2^.

**Figure 4:**
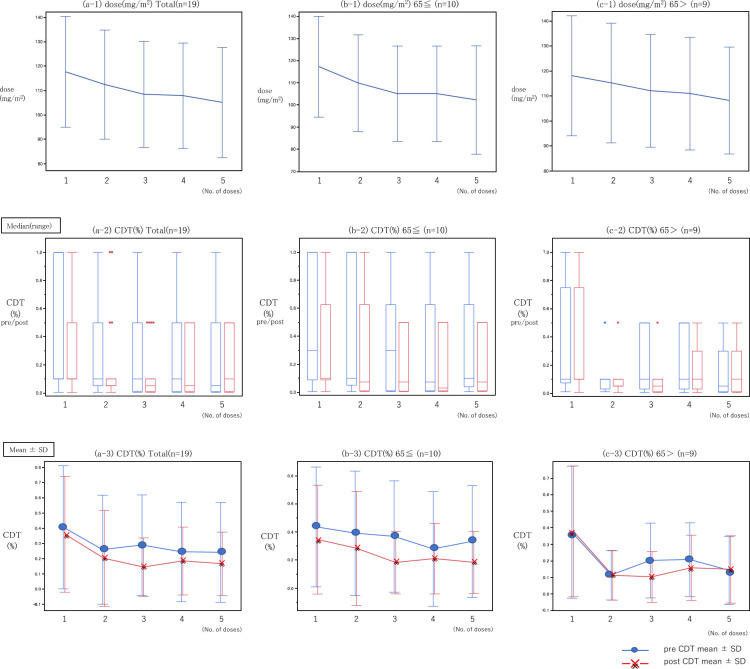
Changes in dose and cold sensation detection thresholds (CDTs) in patients who underwent five menthol tests. (a-1), (b-1), (c-1): Compared with the initial dose (mg/m^2^), each dose was gradually reduced in the subsequent doses, and the values were significantly lower (2nd dose: *P*=0.026, 3rd dose: *P*=0.002, 4th dose: *P*=0.002, 5th dose: *P*=0.002). (a-2, 3): For the CDT value of all cases, the CDT decreased as the number of doses increased, and the third CDT value was significantly lower than that before the first dose (first CDT 0.41→third CDT 0.30, *P*=0.04). (b-2, 3): For the CDT value of cases aged ≥65 years, the CDT decreased as the number of doses increased, and the CDT value tended to be lower than that before the first dose. (c-2, 3): For the CDT value of cases aged <65 years, the CDT decreased as the number of doses increased, and the second CDT value was significantly lower than that before the first dose (first CDT 0.41→second CDT 0.11, *P*=0.04).

The CDT decreased as the number of doses increased, and the third CDT value was significantly lower than the CDT value before the first dose (first CDT 0.41→ third CDT 0.30, *P*=0.04). Comparing the CDT before and after oxaliplatin administration, there were significant decreases in the CDT after administration of the 3rd and 5th doses only (3rd dose: *P*=0.0039, 5th dose: *P*=0.04) (Figure 4, Table 1).

### Endpoint 3

**Figure 5:**
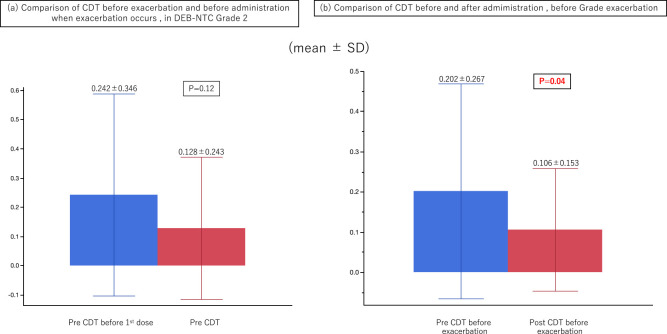
(a) Comparison of the pre-administration cold sensation detection threshold (CDT) before exacerbation and when exacerbation occurred in cases with Neurotoxicity Criteria of Debiopharm (DEB-NTC) grade 2. The CDT tended to be lower when exacerbation occurred, but this change was not significant (*P*=0.12). (b) Comparison of CDT before and after administration, before grade exacerbation. The CDT was significantly reduced in all grade-exacerbated cases, not only in grade 2 (*P*=0.04).

In patients with DEB-NTC grade 2 neuropathy, we compared the pre-administration CDT before grade exacerbation and the pre-administration CDT when exacerbation occurred. There was no significant difference between the two, although there was a tendency toward a lower value when exacerbation occurred (*P*=0.11). When cases of grade exacerbation were examined (grades 0→1, 0→2, and 1→2), the CDT before exacerbation was significantly decreased after oxaliplatin administration (*P*=0.04) (Figure 5).

## Discussion

Oxaliplatin is a key drug that is used in chemotherapy for colorectal cancer. However, although it is used as standard care in adjuvant chemotherapy for unresectable colorectal cancer,^14–16^ peripheral neuropathy is a common adverse effect, so its dose is limited, which is considered to lower its effectiveness.^17^

OPN affects the QOL of patients; thus, it is very important to prevent such neuropathy. To date, for chemotherapy-induced neuropathy, treatments such as Ca^2+^, Mg^2+^, Chinese herbal medicine, and analgesics have been administered prophylactically; however, in clinical trials, none of these treatments have been demonstrated to resolve this problem. In addition to these drugs, opioids and analgesic aids have been used to treat OPN, but similar results have been reported. Duloxetine is currently the only drug that has been shown to reduce neuropathic pain in phase III trials, and is the only treatment recommended in the American Society of Clinical Oncology guidelines.^18^

Methods of administration are also being investigated for the treatment of neuropathic pain. In the OPTIMOX study, in a “stop and go” group in which oxaliplatin was systematically suspended, the worsening of neuropathy was significantly suppressed compared with the continuous administration group, and survival time was not shortened.^19^ These findings suggest that, in clinical practice, it is extremely important to withdraw oxaliplatin at the appropriate time; however, no clear conclusion has been reached regarding when to stop its use. In the CTCAE, which depends on subjective symptoms, the time to reach grade 2 or higher (which is usually be considered the time for withdrawal) varies. This is because CTCAE grading is greatly affected by the personality and treatment goals of each patient. The DEB-NTC, which includes an evaluation of symptom improvement in 7 days, is relatively objective, but because it is also based on each patient’s own opinions, it cannot be considered a truly objective evaluation.

In the present study, we focused on the menthol test that was described in previous studies,^12^ and objectively evaluated OPN by measuring the CDT before and after the administration of oxaliplatin. We also considered the possible utility of the CDT in the menthol test as an evaluation method for chronic disorders by examining changes over time, which were not assessed in previous studies.

Kono et al.^12^ suggested that TRPM activation and neurotoxicity may be correlated, and that acute peripheral neurotoxicity can be determined quantitatively using the CDT—especially in acute neuropathy—in response to cold stimuli.^12^

In the current study, when we compared the CDT before and after the first dose of oxaliplatin, it tended to decrease after administration, but this difference was not significant. Based on reports that older people are more likely to develop OPN, we also took the World Health Organization standard CDT from around the age of 65 years old into consideration. Unlike in a previous study,^12^ no significant difference was observed between subjects who were younger than 65 years old and those who were 65 or older; however, in the group aged 65 and older, the CDT did tend to decrease, thus demonstrating some reproducibility in the CDT changes before and after the first dose in the menthol test (Figure 2).

OPN can be classified into acute or chronic OPN.^17^ Acute OPN is transient (it does not last for more than 7 days), often occurs during treatment, and is characterized by the rapid onset of peripheral paresthesia, resulting in cold hypersensitivity of the limbs and pharynx.^20^ In contrast, chronic OPN is a delayed peripheral neuropathy that occurs after repeated doses of oxaliplatin, and generally manifests as a loss of sensation or numbness, tingling in the limbs, and ataxia.^14,15^ Most patients who receive oxaliplatin appear to develop dose-dependent neurotoxicity.^21^ The incidence of acute neuropathy secondary to oxaliplatin ranges from 80% to 98%.^22^ Chronic neuropathy, in contrast, is expected to occur in approximately 10% to 20% of patients with cumulative doses of oxaliplatin above 750 to 850 mg/m^2^.

In the present study, the menthol test was performed up to five times with oxaliplatin administration, and the dose reduced as the number of times increased (Figures 3 and 4, Table 1). In patients receiving five doses who underwent the menthol test, the mean total dose was 553.79 (340–650) mg/m^2^. The reason for setting the number of doses to five was because allergies, which are a major adverse effect of oxaliplatin, are expected to increase after that point; furthermore, the use of more than five doses may lead to the development of chronic symptoms in clinical practice. Our aim was to evaluate the best timing for discontinuing administration.

When repeating the dose, the CDT tended to decrease, and there was a significant decrease after the third dose compared with before the first dose, suggesting that the CDT tends toward a low value (i.e., hypersensitivity occurs). In addition, when comparing between before and after each dose, the CDT tended to decrease after each dose; this decrease was significant for the 3rd and 5th doses. Similarly, in the comparison by age groups, there was a similar tendency toward a decrease (Figure 4, Table 1).

Our findings also demonstrated that the CDT decreased over time as the total dose of oxaliplatin increased. This finding suggests that the CDT decreased because of the cumulative toxicity of oxaliplatin, which led to acute OPN. However, although a downward trend was observed, if we want to use this information in the future, we will need to evaluate how much it decreases, and precisely how it is related to OPN when used in clinical practice. It will also be necessary to examine the relationship between the degree of OPN and the change in CDT in greater detail, using a larger number of cases.

In the present study, there were no cases with DEB-NTC grade 3 neuropathy, and no cases of irreversible neuropathy or neuropathy that required years to recover from. Hence, the CDT tended to decrease as the number of doses increased, suggesting that oxaliplatin neurotoxicity increases the sensitivity to cold stimuli. Previously, Kono et al.^12^ reported that multiple doses of oxaliplatin significantly increases the baseline CDT and induces cold hypoesthesia. In the current study, the CDT up to the 5th dose showed a downward trend, but we did not further examine the effects of dose. Thus, in the future it may be necessary to investigate how the CDT changes by increasing the number of doses, to be able to apply the menthol test for practical use in the objective diagnosis of OPN.

In the aforementioned self-administered questionnaire for OPN, we examined the relationship between the DEB-NTC score and the CDT, from which we believe we can make relatively objective judgments. There were 12 patients with grade 2 neuropathy, and when we compared the pre-administration CDT before grade exacerbation with the pre-administration CDT when exacerbation occurred, there was a trend toward a lower CDT when exacerbation occurred, although this was not a significant difference. This finding suggests that if the menthol test is performed when exacerbation occurs, it may be able to objectively evaluate the exacerbation of peripheral neuropathy. When we compared the CDT before and after administration, but before exacerbation, the CDT was significantly reduced in all grades of the exacerbated cases, and not only in those with grade 2. This result indicates that neuropathy may possibly be predicted by comparing the CDT before and after administration before exacerbation occurs (Figure 5). Specifically, this might be taken as the timing to discontinue or reduce the drug.

As we demonstrated in this study, the usefulness of measuring the CDT before and after each administration of oxaliplatin is that, by predicting the exacerbation of OPN at an early stage, irreversible OPN and neuropathy that requires a long period of recovery can be avoided. In this way, we believe that treatment can be continued and the therapeutic effect can be improved.

Taken together, our findings suggest that the menthol test can be used to objectively evaluate OPN at an early stage. By appropriately adjusting the dose of oxaliplatin and timing its discontinuation, the QOL of patients can likely be improved, and chemotherapy can be performed more effectively.

The present work was an observational study that confirmed the results of previous studies and observed changes over time, although its statistical detection power was inadequate because there were many missing data. However, the trend was the same as that in previous studies. To support the use of this menthol test in clinical practice in the future, a larger number of cases and more detailed examination are required.

## Conclusion

By performing a menthol test in oxaliplatin-treated patients, it may be possible to objectively predict the exacerbation of peripheral neuropathy at an early stage.

## Figures and Tables

**Table1:** Dose and CDT comparison of patients who underwent 5 menthol tests

Comparison of dose and CDT			
Subjects who underwent 5 menthol tests , dose (mg/m^2^)			
Total (n=19)	Dose comparison with previous dose		Dose comparison with initial dose
1^st^ dose–2^nd^ dose	**P=0.03**	1^st^ dose–2^nd^ dose	**P=0.03**
2^nd^ dose–3^rd^ dose	**P=0.03**	1^st^ dose–3^rd^ dose	**P=0.002**
3^rd^ dose–4^th^ dose	P=0.17	1^st^dose–4^th^ dose	**P=0.002**
4^th^ dose–5^th^ dose	P=0.08	1^st^ dose–5^th^ dose	**P=0.002**
65≤ (n=10)			
1^st^ dose–2^nd^ dose	P=0.05	1^st^ dose–2^nd^ dose	P=0.05
2^nd^ dose–3^rd^ dose	P=0.08	1^st^ dose–3^rd^ dose	**P=0.01**
3^rd^ dose–4^th^ dose	–	1^st^ dose–4^th^ dose	**P=0.01**
4^th^ dose–5^th^ dose	P=0.17	1^st^ dose–5^th^ dose	**P=0.02**
65> (n=9)			
1^st^ dose–2^nd^ dose	P=0.17	1^st^ dose–2^nd^ dose	P=0.17
2^nd^ dose–3^rd^ dose	P=0.09	1^st^ dose–3^rd^ dose	**P=0.05**
3^rd^ dose–4^th^ dose	P=0.17	1^st^dose–4^th^ dose	**P=0.04**
4^th^ dose–5^th^ dose	P=0.17	1^st^ dose–5^th^ dose	**P=0.02**

**Table:** 

Subjects who underwent 5 menthol tests , CDT(%)			
Total (n=19)	CDT comparison with initial CDT		CDT before and after administration of oxaliplatin
1^st^ dose–2^nd^ dose	P=0.06	1st	P=0.18
1^st^ dose–3^rd^ dose	**P=0.04**	2nd	P=0.12
1^st^ dose–4^th^ dose	**P=0.03**	3rd	**P=0.004**
1^st^ dose–5^th^ dose	**P=0.04**	4th	P=0.11
		5th	**P=0.04**
65≤ (n=10)			
1^st^ dose–2^nd^ dose	P=0.36	1st	P=0.17
1^st^ dose–3^rd^ dose	P=0.26	2nd	P=0.13
1^st^ dose–4^th^ dose	P=0.12	3rd	**P=0.02**
1^st^ dose–5^th^ dose	P=0.23	4th	P=0.21
		5th	**P=0.03**
65> (n=9)			
1^st^ dose–2^nd^ dose	**P=0.04**	1st	P=0.66
1^st^ dose–3^rd^ dose	**P=0.03**	2nd	P=0.42
1^st^ dose–4^th^ dose	P=0.08	3rd	P=0.08
1^st^ dose–5^th^ dose	**P=0.03**	4th	P=0.14
		5th	P=0.68
