# Supplementary material for: Establishment of the menthol test as a clinical evaluation method for oxaliplatin-induced neuropathy
Source: Fujita Med J. 2021 Nov 25;8(3):88–95. doi: 10.20407/fmj.2021-011 (PMC9358673; doi:10.20407/fmj.2021-011)
Supplement: Supplementary file 1 — Supplementary Tables [file fmj-8-088-s001.pdf]

Supplemental table

CDT Total(n=19)						
		1	2	3	4	5
Pre	Mean ± SD	0.406±0.404	0.259±0.358	0.289±0.329	0.245±0.326	0.242±0.328
	Median(range)	0.1(0.005-1)	0.1(0.005-1)	0.1(0.005-1)	0.1(0.005-1)	0.05(0.005-1)
Post	Mean ± SD	0.361±0.380	0.202±0.316	0.144±0.192	0.185±0.222	0.166±0.208
	Median(range)	0.1(0.005-1)	0.05(0.005-1)	0.05(0.005-0.5)	0.05(0.005-0.5)	0.1(0.005-0.5)

CDT 65≦ (n=10)						
		1	2	3	4	5
Pre	Mean ± SD	0.436±0.426	0.391±0.442	0.368±0.396	0.279±0.408	0.332±0.398
	Median(range)	0.3(0.005-1)	0.1(0.005-1)	0.3(0.005-1)	0.075±(0.005-1)	0.1(0.005-1)
Post	Mean ± SD	0.346±0.387	0.283±0.405	0.182±0.222	0.210±0.250	0.183±0.222
	Median(range)	0.1(0.005-1)	0.075(0.005-1)	0.075(0.005-0.5)	0.03(0.005-0.5)	0.075(0.005-0.5)

CDT 65> (n=9)						
		1	2	3	4	5
Pre	Mean ± SD	0.373±0.400	0.113±0.150	0.202±0.226	0.207±0.223	0.142±0.205
	Median(range)	0.1(0.01-1)	0.1(0.01-0.5)	0.1(0.005-0.5)	0.1(0.005-0.5)	0.05(0.005-0.5)
Post	Mean ± SD	0.378±0.396	0.112±0.149	0.103±0.154	0.157±0.198	0.148±0.204
	Median(range)	0.1(0.005-1)	0.05(0.005-0.5)	0.05(0.005-0.5)	0.1(0.005-0.5)	0.1(0.005-0.5)

Changes in CDT in patients who underwent 5 menthol test . Mean±SD,Median(range)

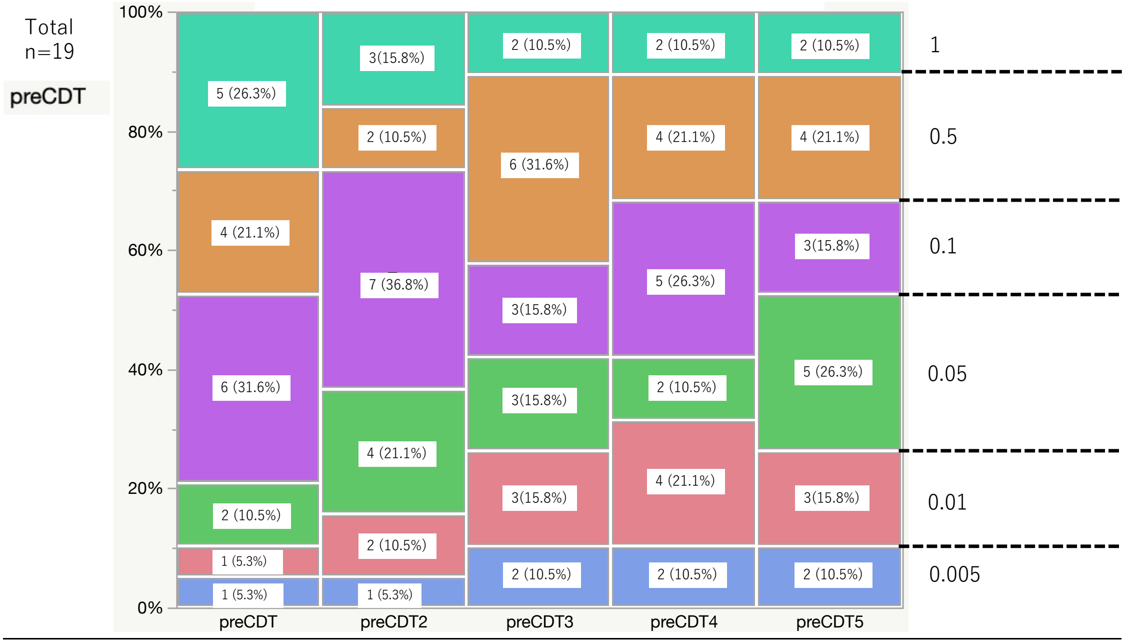

preCDT of all cases (n=19)

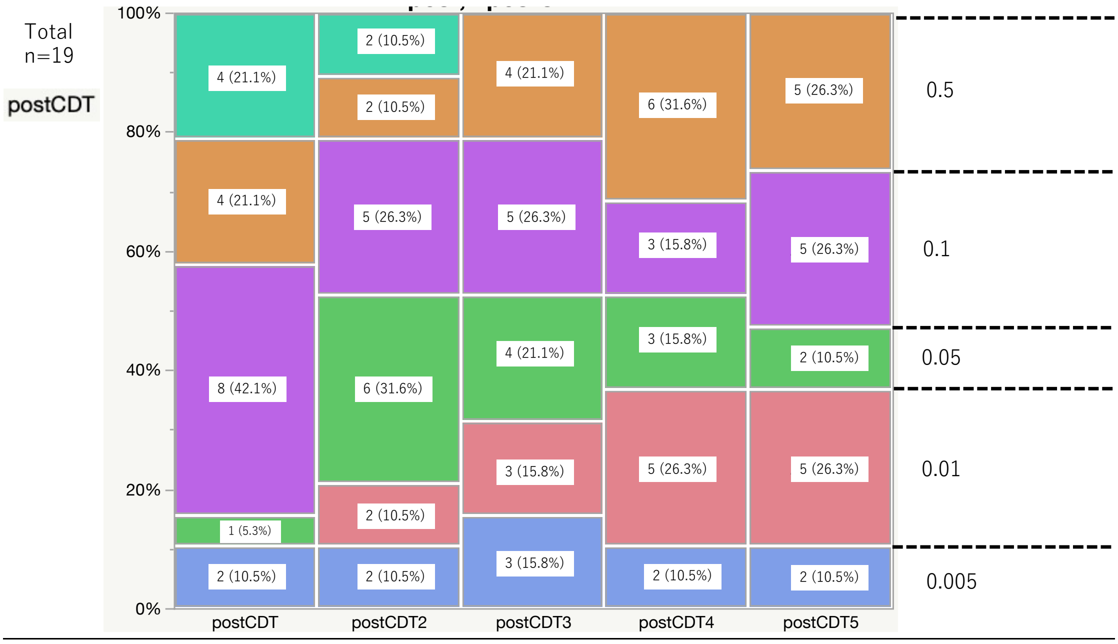

postCDT of all cases(n=19)

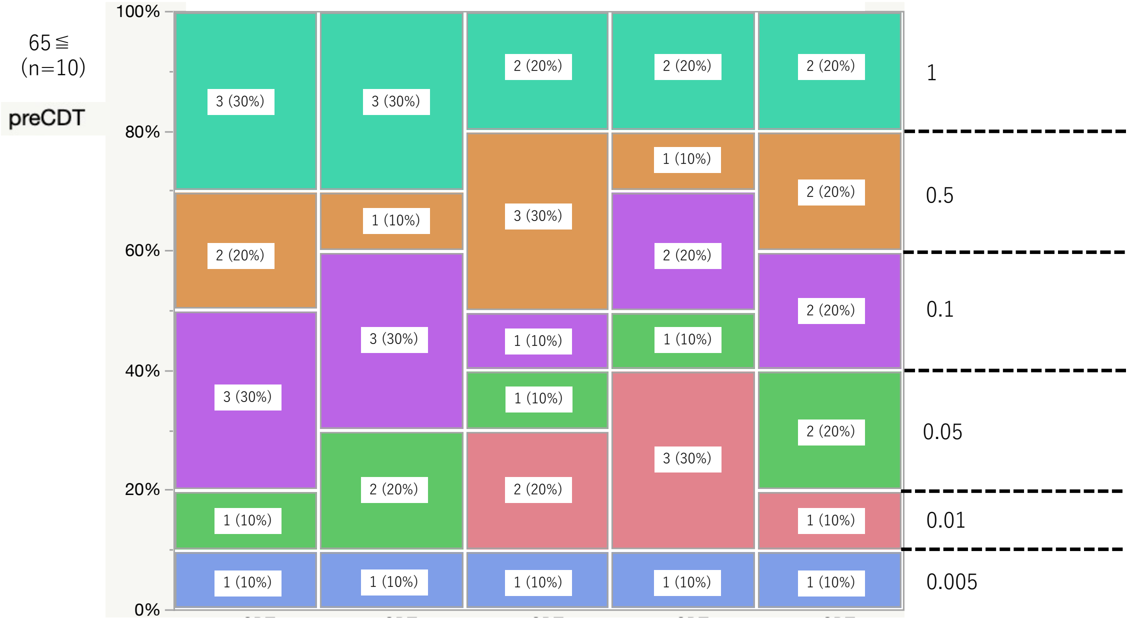

preCDT of 65≦cases

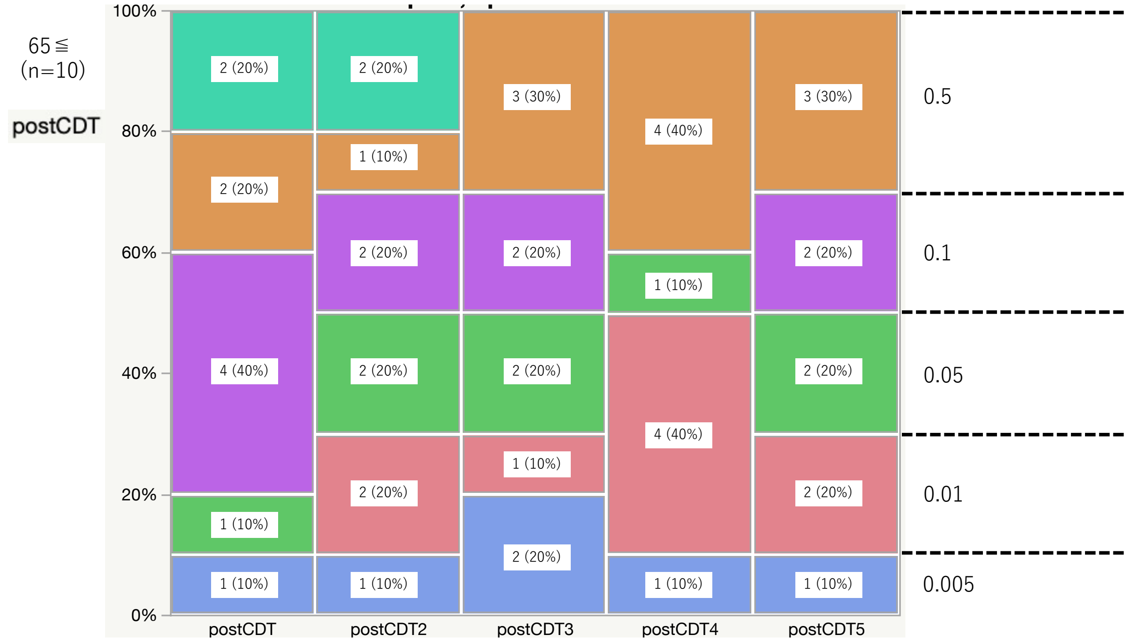

postCDT of 65≦cases

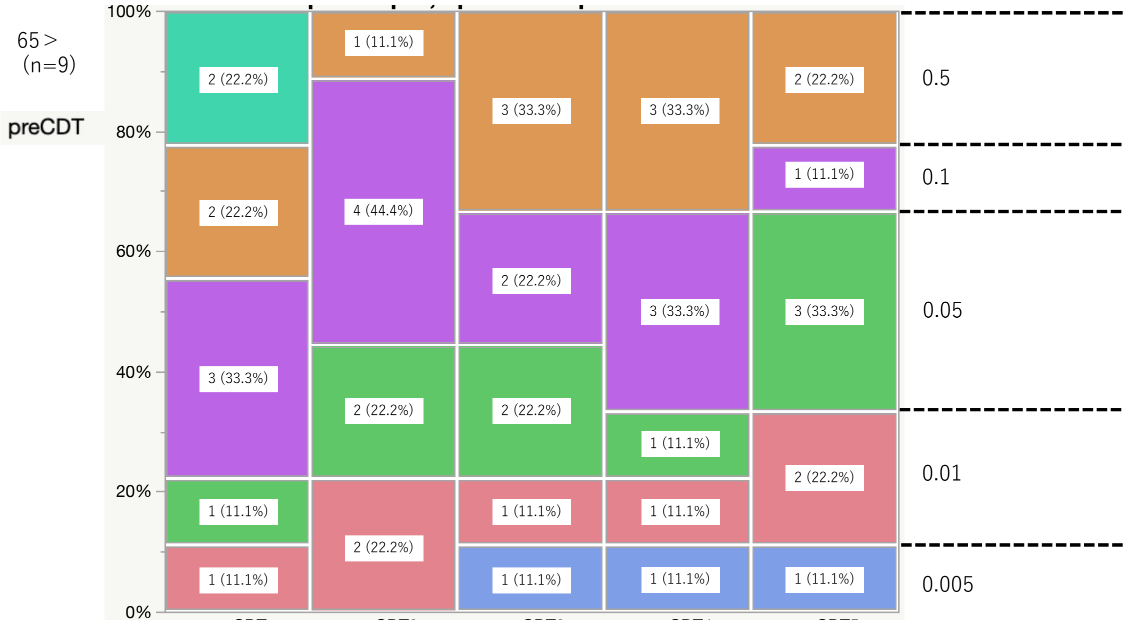

preCDT of 65＞cases

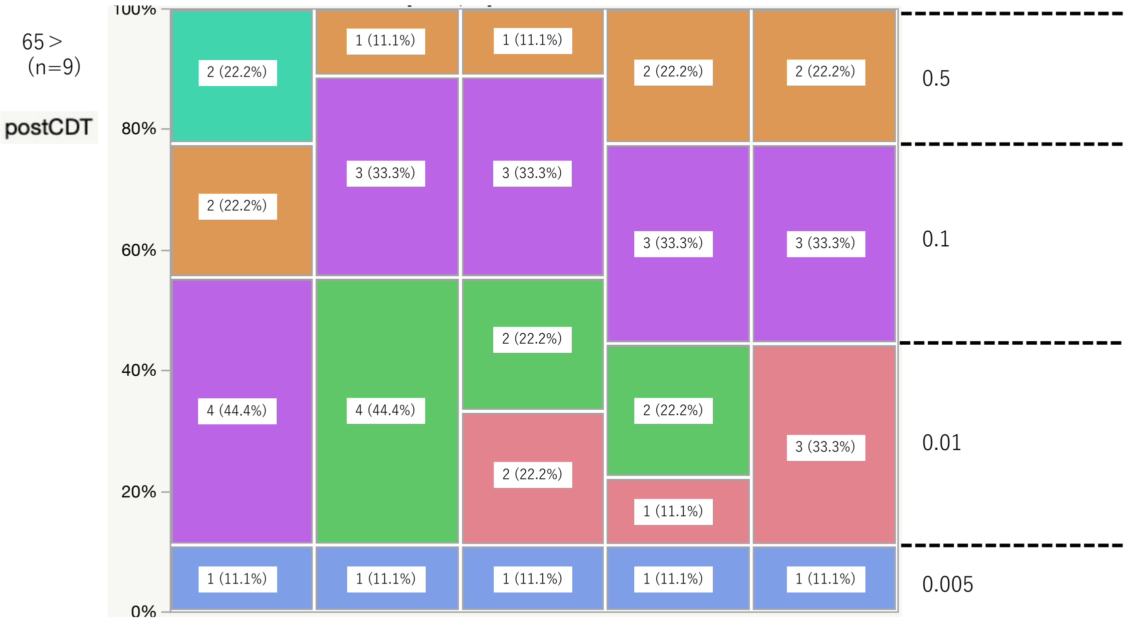

postCDT of 65＞cases
